# Eye-brain connection: an altered profile of spatial attention in myopia

**DOI:** 10.3389/fnins.2025.1593463

**Published:** 2025-05-23

**Authors:** Elie De Lestrange-Anginieur, Feng Pan, Benjamin Thompson, Kin Yau Wong

**Affiliations:** ^1^School of Optometry, The Hong Kong Polytechnic University, Kowloon, Hong Kong SAR, China; ^2^Centre for Myopia Research, School of Optometry, The Hong Kong Polytechnic University, Kowloon, Hong Kong SAR, China; ^3^Research Centre for SHARP Vision (RCSV), The Hong Kong Polytechnic University, Kowloon, Hong Kong SAR, China; ^4^Laboratory of Experimental Optometry, Centre for Myopia Research, School of Optometry, The Hong Kong Polytechnic University, Kowloon, Hong Kong SAR, China; ^5^Centre for Eye and Vision Research (CEVR), Kowloon, Hong Kong SAR, China; ^6^School of Optometry and Vision Science, University of Waterloo, Waterloo, ON, Canada; ^7^Department of Applied Mathematics, The Hong Kong Polytechnic University, Kowloon, Hong Kong SAR, China

**Keywords:** refractive errors, myopia, visuospatial attention, visual field, attention window

## Abstract

**Background:**

Refractive errors represent an important cause of visual impairment, impacting the quality of vision in billions of people across the globe. Degraded visual input may cause individuals with refractive errors to deploy greater attentional resources during visual tasks. We tested the hypothesis that myopia alters the pattern of visual attention.

**Methods:**

Twenty participants (10 near-emmetropes) performed an acuity discrimination task at random visual field locations (eccentricity range: 1–10°; spacing: 1°; polar coordinates of 0°, 90°, 180°or 270°) under conditions of neutral attention (no information on the stimulus position) and focused attention (target presentation in a single meridian), while fully optically corrected. The spatial distribution of attention-related modulation was estimated by the ratio of performance between the focused and neutral conditions across spatial eccentricities using acuity resolution (primary outcome) and reaction times (RT), as measures of attention.

**Results:**

Use of linear mixed models revealed that the enhancing effect of attention follows a cubic spatial profile for acuity and RT, indicating a finite attentional window in myopes and emmetropes with a peak eccentricity at around 4°. Significant dependence of attention modulation on polar coordinate and refractive status was also identified (for both acuity and RT), with larger attention enhancement at the South position, compared to the North location, and lower attention efficiency in myopes, as compared to emmetropes across the visual field. Our modeling of attention-related modulation in acuity further indicated that myopes experience narrower attentional windows, suggesting a reallocation of cognitive resources.

**Conclusion:**

The study is the first, to our knowledge, to provide a detailed spatial profile of attention-related modulation linked to mild to regular myopia, highlighting a differential shape of the focus of attention with refractive status, which demonstrates a redistribution of attention with myopia. This revealed a link between myopia and visual attention, which requires further investigation.

## 1 Introduction

### 1.1 Epidemiology and etiology of myopia

Myopia prevalence has raised important concerns in the scientific community (Dolgin, 2015), as one of the ocular problems that affect most people in the world. East Asia exhibits some of the highest rates worldwide (Holden et al., 2016), followed by North America, with over 80% of 15-year-olds in Singapore, Hong Kong and Taiwan affected by myopia (Rudnicka et al., 2016). Although regions such as Europe and South America, presents low myopia prevalence (Holden et al., 2016), projections indicate that by 2050, nearly half of the global population could become myopic (Dolgin, 2015; Liang et al., 2025). Appearing during the early school years (Lam et al., 2012), myopia generally manifests as a progressive, irreversible over-elongation of the eyeball, which shifts the range of vision to near distances, with blurring at far distances. Although wearing spectacles can normalize myopic vision, an overly elongated eyeball can lead to significant sight-threatening ocular/visual complications (Holden et al., 2015) and changes in certain neural networks in cases of high myopia (Ji et al., 2022; Cheng et al., 2020; Zhang et al., 2020; Zhai et al., 2016; Huang et al., 2016). Despite extensive research, the detailed etiology of myopia and its interaction with the neural systems, remains unknown (Troilo et al., 2019). Supported by abundant animal studies (Wallman and Winawer, 2004), the dominant hypothesis is that myopia is driven by exogenous visual cues, like defocus, present in our environment. These animal studies demonstrate precise structural compensatory changes to the eyeball in response to lens-imposed defocus, implying that myopic changes could represent attempts of the visual system to improve neural image quality (Schaeffel and Swiatczak, 2024). However, this irreversible ocular-based compensation could compete with more flexible neural mechanisms (Horikawa and Kamitani, 2022), such as selective visual attention.

### 1.2 Visual attention and its relevance to myopia

Visual attention (Desimone and Duncan, 1995) is a mechanism supported by a complex neuronal network, which links different brain regions, enabling enhanced visual processing through reflexive or/and volitional prioritization of information (Corbetta and Shulman, 2002; Corbetta, 1998). Among the different types of attention, spatial attention is of special interest for visual acuity (Carrasco, 2018). Studies have shown that attention directed to peripheral retinal regions can enhance peripheral visual acuity under computer-simulated blur (De Lestrange-Anginieur et al., 2021; De Lestrange-Anginieur and Kee, 2020). Such attentional enhancement may also be useful when switching fixation from clear (near) vision to more computationally demanding, blurry (far) vision in myopes. The Zoom lens model (Eriksen and St. James, 1986) proposes an adaptative attentional solution, whereby attention scales to the task/stimulus demands. This attentional scaling could take different spatial shapes, producing either a narrowing or broadening of the attentional window, i.e., the region over which attentional resources are deployed and observable attentional effects take place (Reynolds and Heeger, 2009; Yeshurun, 2019). Narrowing of the attentional window has been demonstrated to boost various behavioral tasks (Lawrence et al., 2020), including spatial resolution. However, whether myopes use this strategy to overcome blurry visual inputs during episodes of uncorrected vision (before refractive correction or when removing eyeglasses) remains elusive.

### 1.3 Attentional component in myopia

Previous studies have suggested visuospatial attentional differences between myopes and emmetropes (Turatto et al., 1999; Mascetti et al., 2001; McKone et al., 2008; Kerber et al., 2016). Turatto et al. (1999), explored the effect of spatial attention on myopia using a Posner paradigm, measuring reaction times of peripheral targets situated at 8° and 16°, and found greater attention deficit in the peripheral visual field of myopes compared to emmetropes. McKone et al. (2008) investigated the hypothesis of a narrowing of attention in myopia using an inverted half-face task paradigm, in which participants were required to focus rapidly on a half-face feature while ignoring the other half (the distractor) under low (misaligned halves) and high interference (aligned). The task revealed a positive correlation between the level of refractive errors and the ability of participants to focus on the half-face, supporting enhanced focal attention in myopes under demanding visual tasks, as compared to emmetropes. More recently, it was also reported that myopes tend to suffer more from deploying their attention around the fovea (Kerber et al., 2016) when performing a peripheral detection task, which could imply attentional deficits of myopes in extrafoveal regions. However, this attentional interference was only present in the retinal mid-periphery, but not in the near-periphery, raising the question of whether spatial attention is globally decreased with myopia and its focus narrowed around the fovea.

### 1.4 Aim and hypothesis

Despite accumulating evidence of peripheral attentional deficits in myopes, the spatial profile of attention that underlies refractive-related differences and its root, remain unstudied. While interest has grown in quantifying the focus of attention (Yeshurun, 2019), the attentional window in myopes is currently unknown, and only a few studies (Koenig-Robert and Van-Rullen, 2011; Gobell et al., 2004; Tse, 2004) have attempted to characterize the detailed profile of spatial attention in normal participants (i.e., without specified refractive errors). For example, Tse (2004) employed a change blindness paradigm, exploiting the need for attention to detect local changes under full-field change, to develop the first fine-grained attentional map based on detection accuracy. Concurrently, Gobell et al. (2004) proposed to explore attention modulation in a visual search paradigm, where carefully engineered grating cue patterns were utilized to manipulate the distribution of spatial attention. Later, Koenig-Robert and Van-Rullen (2011) used a cue-based paradigm to construct spatiotemporal maps of attention-related modulations of contrast detection within the central visual field ±10° (spatial sampling: 2°) for various cue delays, revealing a temporally stable attention profile marked by distinct modulation peaks. In all these studies, attention modulation was derived from the comparison of two different attention states, with one being neutral. Building on this foundation, the present study utilized a spatial uncertainty-based attention paradigm to determine the spatial distribution of attention modulation around the fovea in myopes and emmetropes. By manipulating spatial uncertainty to control attentional allocation (Fortenbaugh et al., 2017), this approach avoided external cue/distractor stimuli used in previous mapping paradigms (Tse, 2004), which could interfere with target processing. We hypothesized that myopes would exhibit a narrowing of the attentional profile at the center of vision compared to emmetropes. The results revealed detailed spatial profiles of attention modulation in the center of vision for both refractive groups, indicating clear differences in endogenous spatial attention between myopes and emmetropes.

## 2 Methods

### 2.1 Participants

This randomized case–control study recruited 20 young Chinese adults (age 18–26 years, 50% Females) divided into 10 near-emmetropes and 10 myopes. Participants were recruited through advertisements on the university campus and through personal referrals among optometry students. Each potential participant attended an initial visit for an eye examination, during which the following assessments were conducted on each eye:

1. Best corrected distance visual acuity (VA), measured using a Snellen letter chart with crowded letters.
2. Refractive error, assessed through cycloplegic subjective refraction using two drops of 1% Tropicamide to temporarily paralyze the eye’s accommodation system and ensure accurate measurement.
3. Visual field, evaluated with the Humphrey Field Analyzer (HFA) using a 24–2 threshold and SITA Fast strategy test.
4. Collection of basic demographic information, such as age and gender.
5. Training on a Tumbling E acuity task.

Participants who met the inclusion criteria were invited to return for a subsequent visit to participate in the main experiments. The selected participant baseline data are summarized in Table 1.

**Table 1:** Demographic and clinical characteristics of participants.

**Participants**	**Rx status**	**cSER**	**Cylinder**	**Cylinder axis**	**VA (logMAR)**	**Visual field index (%)**	**Dominant eye**	**Age**	**Gender**
1	Emmetrope	0.5	−0.50	70	−0.1	98	OS	24	F
2	Emmetrope	0.25	−0.50	45	−0.1	100	OS	24	F
3	Emmetrope	0	0.00	180	−0.1	100	OD	22	M
4	Emmetrope	−0.1	−0.25	160	−0.1	100	OD	23	F
5	Emmetrope	−0.1	−0.25	180	−0.1	100	OS	23	F
6	Emmetrope	0.88	−0.75	180	0	99	OS	19	M
7	Emmetrope	−0.6	−0.75	166	−0.1	100	OS	22	F
8	Emmetrope	−0.8	−0.50	95	−0.1	99	OS	22	F
9	Emmetrope	−0.3	0.00	180	−0.1	100	OS	22	M
10	Emmetrope	−0.1	−0.25	180	−0.1	100	OD	22	F
11	Myope	−4.6	−0.75	60	−0.1	99	OD	23	M
12	Myope	−4.1	−0.75	178	0	100	OS	22	M
13	Myope	−3.1	−0.75	150	−0.1	99	OD	21	F
14	Myope	−3.1	−0.75	5	−0.1	100	OS	26	M
15	Myope	−5.8	−0.50	5	0	100	OD	20	F
16	Myope	−2.6	−0.75	165	−0.1	99	OS	22	M
17	Myope	−4	0.00	180	−0.1	100	OS	21	M
18	Myope	−5.5	−0.50	175	0	98	OD	19	F
19	Myope	−1.3	−0.50	180	−0.1	100	OD	22	M
20	Myope	−3	0.00	180	−0.1	100	OS	22	M

The eligibility criteria were: (1) 18–26 years (2) at least 20/20 best corrected VA in both eyes (3) + 1.00D > cycloplegic spherical equivalent refraction (cSER) > −6.00 D. The exclusion criteria included (1) Cylinder >1.00 DC, history of ocular surgery, other eye diseases, or any binocular problems. Myopic participants were identified by their cSER within the range of −1.00D to −6.00D, while near-emmetropes had a |cSER| of <1D. Selecting this age group improved compliance to the long duration of the test and task demand. Our sample size was primarily determined to ensure the detection of a statistically significant effect of attention on visual performance (*p* < 0.05), based on prior research on covert spatial endogenous attention (De Lestrange-Anginieur et al., 2021). Using G*Power, it was further estimated that this sample size would provide over 80% statistical power (*α* = 0.05) to detect a moderate-to-large effect size (d = 0.67, Cohen, 1988) between myopes and emmetropes in a mixed ANOVA analysis. The experimental procedures were approved by the human ethics committee of The Hong Kong Polytechnic University (HSEARS20230502003), and the research was conducted according to the principles expressed in the Declaration of Helsinki. Informed consent was obtained from each participant.

#### 2.1.1 Visual test

##### 2.1.1.1 Field performance task

**Figure 1:**
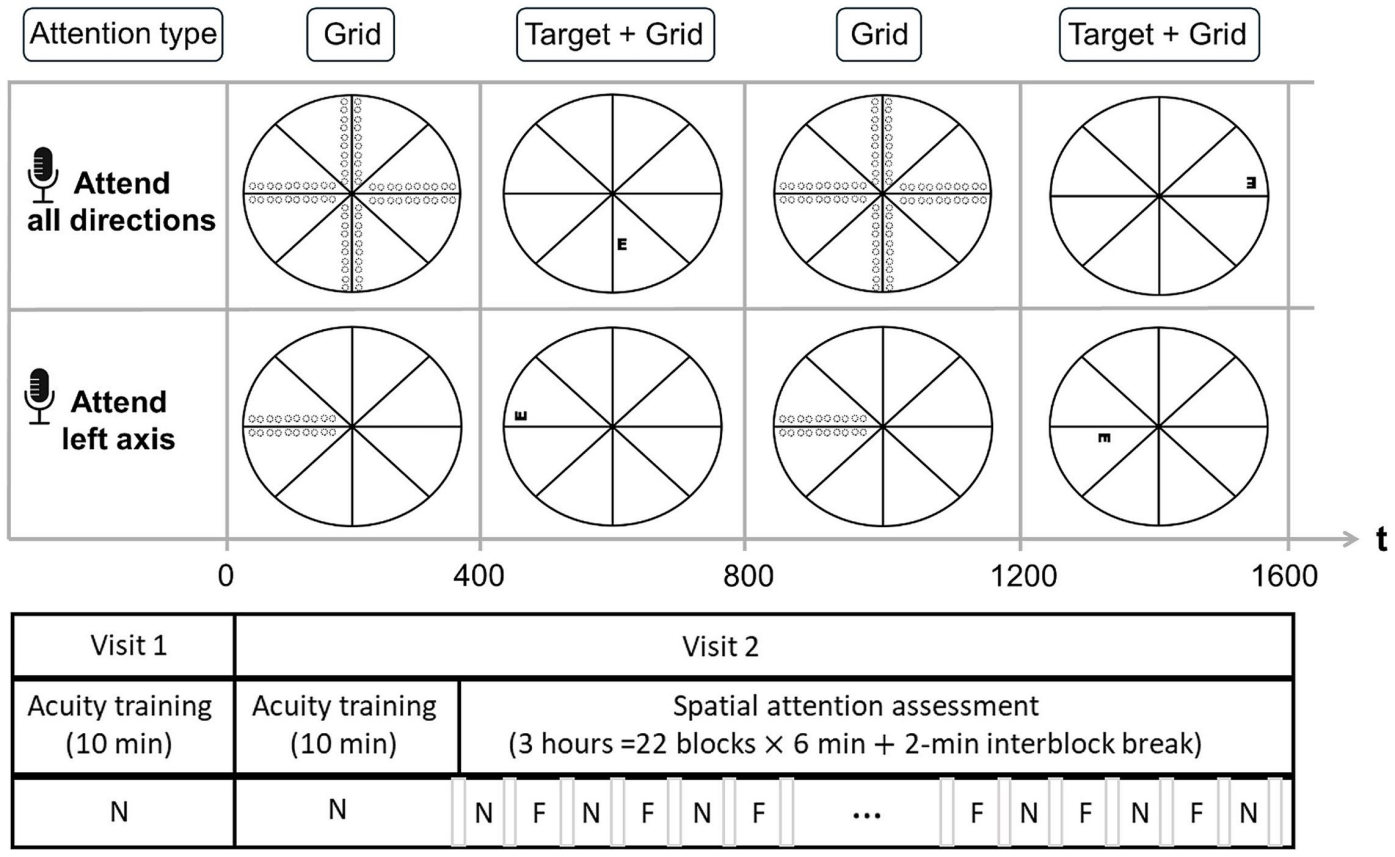
Schematic representation of the experimental design. (Top panel) Neutral Attention Condition (N): Participants maintained attention across all radial grid orientations, with the Tumble E target presented randomly at any potential location. (Middle panel) Focused Attention Condition (F): Participants directed their attention to a single grid axis orientation (leftward orientation shown), with the target appearing only along this axis. (Bottom panel) Test Structure: Following two consecutive acuity training tests, participants completed repeated blocks of the Neutral (N) and Focused (F) spatial attention conditions. The order of conditions (N vs. F first) was counterbalanced across participants. A brief pause separated each block. Dotted circles represent the potential target locations within each condition.

Throughout the study, participants performed a 4 alternative-forced choice Tumble E acuity task on a display (Dell LCD monitor, working distance: 1 m; full visual angle: 22°, background color: black, background luminance: 1 cd/m^2^) under full refractive correction, monocular conditions using their dominant eye (non-dominant eye patched), and without eye dilation. The Black-on-White Tumble E target (duration: 0.4 s) was set with a high-contrast (Peak contrast: 100 cd/m^2^). The target exhibited a gradual onset and offset, featuring a triangular contrast modulation (peak at 0.2 s) As in Rosenberg et al. (2013), this design minimized abrupt stimulus-driven attentional capture (Yantis and Jonides, 1990) and its potential interaction with endogenous attention (Hopfinger and West, 2006; Theeuwes, 1991), ensuring that the observed effects primarily reflect sustained, voluntary attention rather than transient, stimulus-driven responses. The visual target was tested at different locations of the visual field (10 eccentricities X 4 polar coordinates [Leftwards and rightwards, Horizontal, upward and downward vertical] = 160 locations, depicted in Figure 1) to assess visual field acuity.

The eccentricities tested ranged from 1° to 10° with a separation of 1°. Targets were presented laterally to the cross with an edge-to-edge spacing equal to the size of the target to prevent lateral interactions. The size of targets was controlled via a 1-up-1down staircase method, converging to 50%. Step size was set to 3 arcmin for stimuli situated at 1° and adjusted via M-scaling to match the eccentricity-dependent threshold size of the target (Virsu and Rovamo, 1979). Participants were instructed to report the orientation of the E (up, down, left, or right) as fast as possible by pressing a button on a keyboard. For each tested condition, reaction times were taken as the average duration between target onset and response across all trials. A beep was issued whenever responses took place more than 800 ms after stimulus onset. To ensure participants maintained the same strategy and to minimize learning effects, no feedback was provided about the correct orientation of the target. A radial grid (Luminance: 100 cd/m^2^; color: white, width: 1 arcmin, length: 22°) was used to help participants maintain constant fixation throughout the test. An eye tracker (Tobbi TX300) monitored fixation, emitting a warning signal whenever eye movements were detected (radius > 1°).

##### 2.1.1.2 Training/practice tasks

The Tumble E acuity task was conducted in two separate sessions, performed on different days, to allow prior familiarization with the acuity task. In the first session, participants performed a 10-min acuity task across the visual field, with the target size being controlled via an eccentricity-dependent staircase. The 10-min preparatory acuity task was repeated on a separate day before the main test started to ensure optimal performance. The acuity threshold estimated in this preparatory task was used to determine the initial size of the target for the main attention test for each participant and spatial eccentricity.

##### 2.1.1.3 Attention tasks

After training, participants performed the main acuity task under two explicit manipulations of attention: “focused attention” and “neutral attention.” In the focused attention condition, an auditory cue instructed participants to focus their attention along a specific axis of the central cross, whereby all the targets were presented. In the neutral attention condition, an auditory cue instructed participants to focus their attention along all the axes of the central cross, while the targets were presented. Thus, the two attention conditions differed only in the spatial distribution of the tested targets, which created distinct levels of spatial uncertainty. To allow full and continuous deployment of spatial attention, each attention condition was tested in separate blocks of trials. Neutral and focused blocks were presented successively so that the same attention conditions were not repeated between blocks. The sequence of neutral and focused blocks was counterbalanced between participants to ensure no bias. Each block of trials (≥180 trials) lasted 6 min spaced by an intervening pause (~2 min). For both neutral and focused attention tests, a separate staircase was run for each eccentricity, polar coordinate, and meridional orientation of the target to account for potential performance and attentional asymmetry, yielding a total of 80 staircases (10 Eccentricities X 4 Polar coordinates X 2 Meridional orientations of the target) per attention condition, all the staircases being interleaved. The attention test comprised a total of 3,520 trials per participant (total duration: 3 h).

##### 2.1.1.4 Refractive correction

It is well known that off-axis aberrations, like field curvature/ astigmatism, vary quadratically/ cubically with eccentricity, causing only small progressive refractive error changes at lower eccentricities (before gaining in magnitude at higher eccentricities). Given the small visual eccentricity tested in this study, we made the choice of using trial lenses, rather than using advanced correction (e.g., wide-angle adaptive optics simulator) because of the important difficulty of maintaining optimal alignment and correction of the patient throughout a long testing duration. To confirm the negligible effect of the trial lenses, a ray-tracing simulation of the trial lenses used to correct the participants was performed. It confirmed that the variation of spherocylindrical errors introduced by trials lenses within +/−10 degrees were sufficiently small (Spherical equivalent <0.25D, i.e., below clinical significance), to ensure similar performance of correction across eccentricities, and so neglect a potential effect of these residual blurs on acuity, and its derived attention effect. Therefore, the main optical contribution in this study was the eye’s relative peripheral refraction.

## 3 Statistical analysis

### 3.1 Outcome measurement

Performance thresholds were calculated as the average of the minimum angle of resolution (MAR) and RT across all trials for each tested condition. Attention effects (AE) were quantified as the logarithm of the ratio between neutral and focused performance thresholds.

### 3.2 Modeling attention-related effects

To examine the detailed spatial profile of attention effects, attention-related modulations were modeled with first, second, and third-degree polynomial terms of eccentricity, polar coordinate, target orientation, and refractive status as fixed effects with (Model 1) or without adjustment (Model 2) for the corresponding baseline neutral performance (acuity/RT). A full model that included all fixed effects and interactions was initially tested, followed by the development of a reduced model. Model reduction was conducted through forward selection using the second-order bias-corrected Akaike information criterion (AICc) index, designed for small sample sizes, for all possible combinations of the independent variables. This selection enabled a minimalistic model enhancing model fitting, as compared with the full model, and focusing on variables influencing attention effects.

### 3.3 Modeling the discrete effect of attention

To further examine the discrete effects of attention, a linear mixed model was fit with performance threshold (Model 3) and attention effects (Model 4), respectively, set as the dependent variable. In both models, first, second, and third-degree polynomial terms of eccentricity, polar coordinate, target orientation, and refractive status were set as fixed effects, along with all pairwise interaction terms among these variables. Additionally, Model 3 also incorporated attention status (i.e., neutral vs. focused).

### 3.4 Covariance structure

A subject-specific random intercept was incorporated into all models, and the covariance matrix of the error terms of observations from a participant was set to follow the spatial power covariance structure, in which the distance between any two observations (Model 1–2, 4: eccentricity, polar coordinate along the x-axis, polar coordinate along the y-axis, and orientation) was defined as the Euclidean distance between the corresponding standardized parameter value associated with those observations, in a four-dimensional space (eccentricity, polar coordinate along the x-axis, polar coordinate along the y-axis, and orientation) for Models 1,2, and 4 and in a fifth-dimensional space (eccentricity, polar coordinate along the x-axis, polar coordinate along the y-axis, orientation, and attention status) for Model 3.

### 3.5 Correlation between refractive errors and attention effect

Pearson correlations were computed between refractive errors and attention effects for each eccentricity condition to determine the association between the degree of myopia and attention.

### 3.6 Participant outlier removal

In all fitted models, the residual distributions were checked via QQ-plots to ascertain the normality assumption. One outlying myopic participant who showed a grand average attention effect which deviated from the median of the group grand average attentional effects by more than +/−3.5 median absolute deviation was removed from the analysis.

Results are reported as means with standard errors.

## 4 Results

Attention effects were tested with both full (depicted in Supplementary materials) and reduced models (Table 2).

**Table 2:** Forward model selection based on AICc for attention effects.

Order of selection	Attention-related modulations of acuity resolution			
	Model 1		Model 2	
	Included variables	AICc	Included variables	AICc
1	Baseline neutral acuity	−1404.532	Polar coordinate	−1194.002
2	Polar coordinate	−1412.401	Eccentricity	−1192.046
3	Eccentricity	−1587.477	Eccentricity_sq	−1194.064
4	Eccentricity_sq	−1597.661	Eccentricity_cub	−1217.311
5	Eccentricity_cub	−1609.901	Refractive status + Refractive status by Eccentricity	−1218.700
6	Eccentricity*Polar coordinate	−1,628.92		
7	Refractive status + Refractive status by Eccentricity	−1635.152		
Full model		−1571.122		−1159.951

Order of selection	Attention-related modulation of reaction times			
	Model 1		Model 2	
	Included variables	AICc	Included variables	AICc
1	Baseline neutral RT	−4,746.17	Polar coordinate	−3,854.17
2	Polar coordinate	−4,765.44	Eccentricity	−3,852.70
3	Eccentricity	−4,779.66	Eccentricity_sq	−3,853.93
4	Eccentricity_sq	−4,779.66	Eccentricity*Polar coordinate	−3,853.08
5	Refractive status + Refractive status*Polar coordinate	−4,783.59	Eccentricity_sq*Polar coordinate	−3,870.67
6	Eccentricity_cub	−4,788.15		
7	Orient + Orient*Eccentricity	−4,793.03		
Full model		−4729.156		−3814.47

Reduced models of attention effect (Models 1 and 2) improved the model fitting, as compared to full models, highlighting parameters with significant influence on attention effects. The observations obtained from reduced models are described below.

### 4.1 Attentional modulation of acuity

#### 4.1.1 Cubic spatial profile

**Figure 2:**
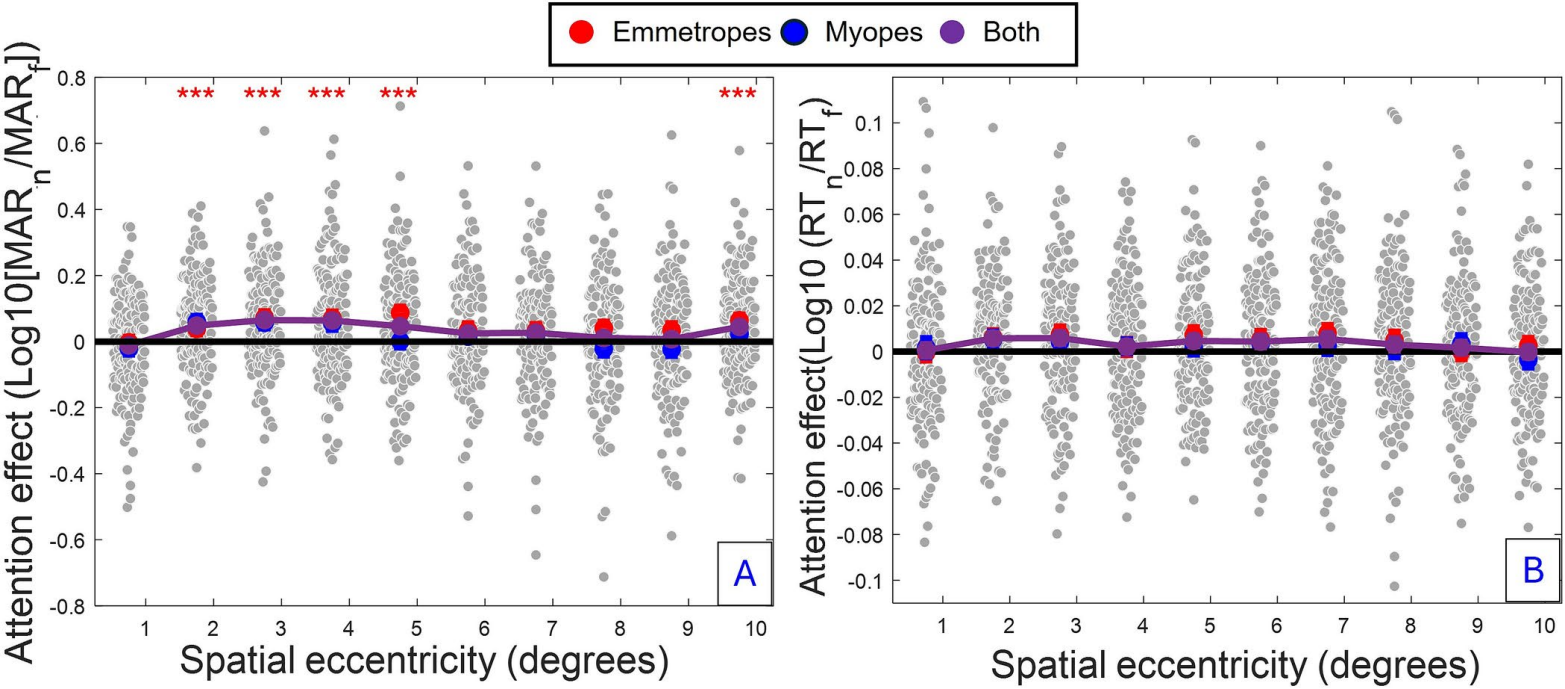
Spatial profile of attention modulation for acuity and RT. **(A)** Acuity: Group-averaged attention enhancement (difference between focused and neutral conditions) (i.e., average across all conditions and participants) as a function of eccentricity **(B)** RT: Same analysis for reaction time. *Grey dots*: individual participant data, *Blue dots:* Myopes (Mean ± SE). Red dots: Emmetropes (Mean ± SE). Purple dots: Combined group means. Red asterisk: Significant attention enhancement (*p* ≤ 0.001, Model 3) associated with spatial eccentricities of the visual field having the highest attention enhancement.

The reduced Model 2 shows a cubic slope profile of the attention modulation [linear eccentricity terms: *F*_(1, 743.959)_ = 23.002, *p* < 0.001; quadratic eccentricity terms: *F*_(1, 814.664)_ = 4.155, *p* < 0.05; cubic eccentricity terms: *F*_(1, 1182.039)_ = 25.586, *p* < 0.001], demonstrating a complex spatial distribution of attention (depicted in Figure 2). The strong, negative effect of linear eccentricity (*β* = −0.065716, SE = 0.013043, *p* < 0.001) indicated a linear decrease trend of attention away from peripheral visual regions. The negative effect of quadratic eccentricity (*β* = −0.011128, SE = 0.005459, *p* < 0.05) coupled with the positive effect of cubic eccentricity (*β* = 0.033179, SE = 0.006559, *p* < 0.001) indicated a peak of attention modulation followed by a trough. Using a full model of acuity (Model 3) for each separate eccentricity, peaks (Eccentricity, 2–4°: *p* ≤ 0.001; 5°: *p* = 0.005, 10°: *p* ≤ 0.05) and troughs (Eccentricity, 1°: *p* > 0.1; 6–9°: *p* > 0.1) of attention modulation were found, highlighting distinct regions of enhancement (i.e., regions 2–5° and 10°) and attenuation of attention (i.e., regions 5° and 6–9°).

#### 4.1.2 Attentional modulation asymmetries

**Figure 3:**
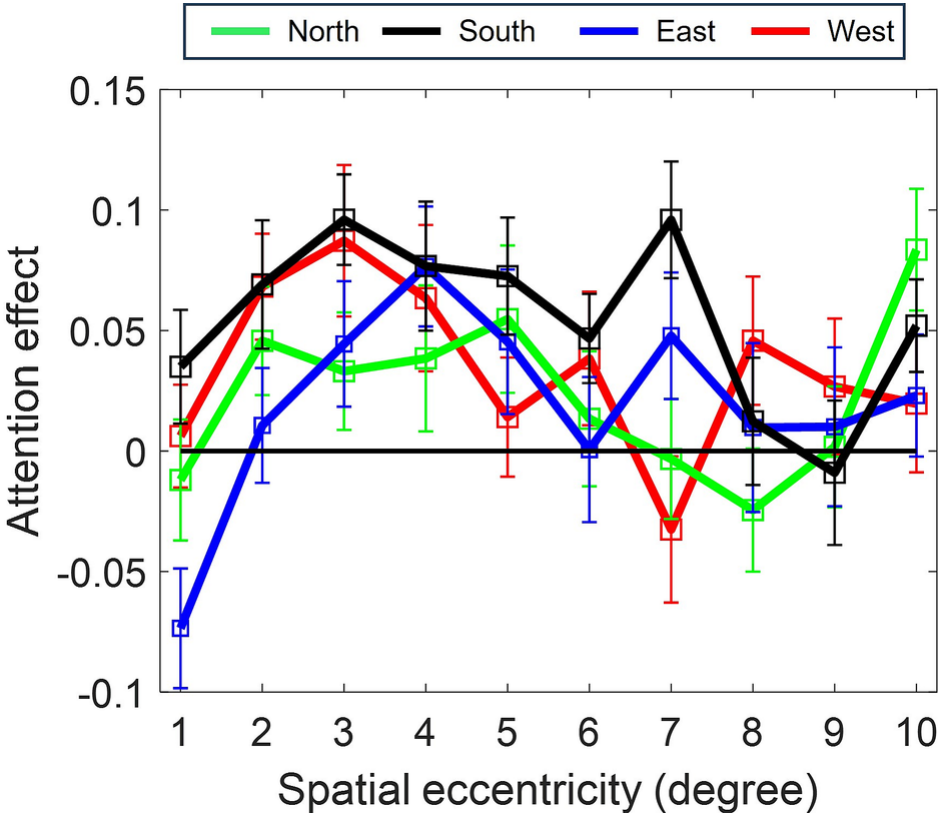
Polar asymmetry in attention enhancement (acuity) across cardinal positions (North, South, East, West). Profile of spatial attention effect for each cardinal position. Greatest enhancement occurs in the *South* position (*p* ≤ 0.001, Model 1–2). Attention modulation increases from the foveal region (1°) to a peak at 4–5° eccentricity before declining for all the cardinal positions.

The effect of attention differed across the location of the visual field (depicted in Figure 3), with superior attention enhancement in the South quadrant (inferior retina), as compared to the North (superior retina) (*β* = −0.030896, SE = 0.013226, *p* < 0.05) and East (*β* = −0.036301, SE = 0.013188, *p* > 0.01) and West (*β* = −0.021286, SE = 0.013188, *p* = 0.1) quadrants.

#### 4.1.3 Attentional differences related to mild to regular myopia

Adjustment for baseline neutral acuity substantially improved the model (see Table 2, Δ AICc = 411.269). A significant positive correlation between baseline neutral acuity and attention effects was found (*β* = 0.015713, SE = 0.000663, *p* < 0.001), suggesting an influence of visual processing performance on attentional efficiency. The adjusted model shows a strong, significant main effect of refractive status by eccentricity [*F*_(1, 196.678)_ = 8.928, *p* = 0.003], providing evidence of the influence of myopia on the spatial profile of attention. Specifically, the near-emmetropes group displayed a lower linear decrease of attention effect with eccentricity compared to the myopic group (*β* = 0.029489, SE = 0.009869, *p* = 0.003).

The significant effect of refractive status on attention effect [Model 4; *F*_(1, 16.999)_ = 4.945, *p* < 0.05] also indicated that the difference in attention effect between myopes and emmetropes was most significantly pronounced around 5° eccentricity. At that eccentricity, a significant positive correlation between the degree of refractive errors at 5° (Pearson correlation: R = 0.264, *p* = 0.001) was also observed, but no correlation was found between refractive errors versus attention at the other eccentricities nor overall attention effects (R = 0.083, *p* = 0.001). In the adjusted model, the effect of attention varied not only with the position of the visual field [*F*_(3, 313.763)_ = 11.777, *p* < 0.001], but also its interaction with the linear term of eccentricity [*F*_(3, 515.366)_ = 8.589, *p* < 0.001]. More specifically, the horizontal cardinal positions displayed a significantly larger increase of attention with the linear term of eccentricity as compared to the South quadrant (East quadrant: *β* = 0.057341, SE = 0.012969, *p* = 0.001; West quadrant: *β* = 0.027922, SE = 0.012944, *p* < 0.05), whilst there was no significant difference in the North quadrant (*β* = 0.002483, SE = 0.013156, *p* > 0.1) compared to the South quadrant. This indicated a potential horizontal-vertical asymmetry in the spatial profile of attention. However, there was no refractive status by eccentricity by polar coordinate interaction, suggesting a similar difference in the pattern of attention of emmetropes versus myopes across polar coordinates.

#### 4.1.4 Model prediction about attention

The reduced model provided a relatively simple model for the profile of attention-related modulation (AE) as shown below:


$$\begin{array}{c} \mathrm{AE}=-0.258591–0.183055\times E-0.019271\times E^{2}\;\\ +0.022921\times E^{3}-0.026419\times [\theta =90^{{}^{\circ}}]+0.039545\times [\theta =0^{{}^{\circ}}]\\ +0.042212\times [\theta =80^{{}^{\circ}}]+0.052144\times [\mathrm{RS}=\text{Emmetrope}]\\ +0.029489\times E\times [\mathrm{RS}=\text{Emmetrope}]-0.002483\times E\times [\theta =90^{{}^{\circ}}]\\ +0.057341\times E\times [\theta =0^{{}^{\circ}}]+0.027922\times E\times [\theta =180^{{}^{\circ}}]\\ +0.015714\times \mathrm{MAR}\;(E,\theta ) \end{array}$$

Where the first-, second-, and third-degree polynomial terms of eccentricity E, E^2^, E^3^, respectively; *θ* is the polar coordinate of the target, RS is the refractive status of the participant, MAR is the acuity threshold.

**Figure 4:**
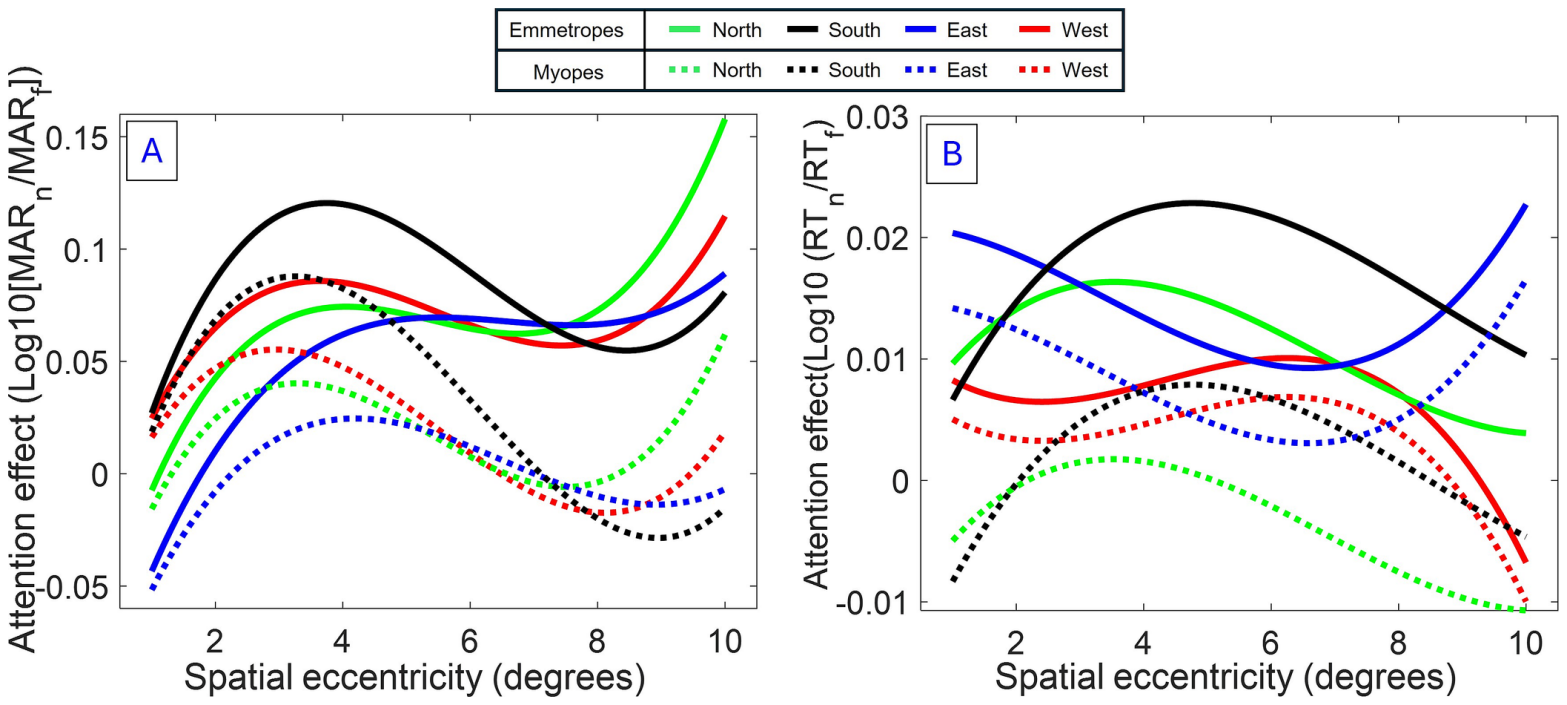
Modeled spatial attention profiles for myopes and emmetropes. **(A)** Acuity: Fitted trajectories of attention modulation as a function of eccentricity (Model 1). **(B)** RT: Same analysis for reaction time. Both measures indicate an attentional window centered around 4°, modulated by polar coordinate and refractive status. Interestingly, for acuity (primary measure), an increasing lowering of attention enhancement with eccentricity was observed in myopes, causing a narrowing of attention.

The model revealed an increasing lowering of attention enhancement with eccentricity in myopes compared to emmetropes, resulting in (i) a shrinkage of the region of attention enhancement -or attentional windows, (ii) a negative effect of attention outside the attentional windows -or surround inhibition- for myopes, and a slight shift in the peak of attention in myopes, compared to emmetropes (see Figure 4).

#### 4.1.5 Attentional modulation of RT (secondary outcome)

A similar reduced model was obtained for attention-related modulation in RT, the secondary outcome, in model 1, as shown below:


$$\begin{array}{c} \mathrm{AE}=-0.404254+0.005102\times E-0.003559\times E^{2}\\ -0.002132\times E^{3}-0.005978\times [\theta =90^{{}^{\circ}}]\\ +0.009520\times [\theta =0^{{}^{\circ}}]+0.005474\times [\theta =180^{{}^{\circ}}]\\ +0.014952\times [\mathrm{RS}=\text{Emmetrope}]+-0.011743\\ \times [\theta =90^{{}^{\circ}}]\times [\mathrm{RS}=\text{Emmetrope}]\\ -0.000353\times [\theta =0^{{}^{\circ}}]\times [\mathrm{RS}=\text{Emmetrope}]\\ -0.008764\times [\theta =180^{{}^{\circ}}]\times [\mathrm{RS}=\text{Emmetrope}]\\ +0.001866\times E\times [\alpha =90^{{}^{\circ}}]-0.004085\times E\\ \times [\alpha =0^{{}^{\circ}}]+0.623750\times \mathrm{RT}\;(E,\theta ) \end{array}$$

Where *α* is the meridional orientation of the target, and RT is the baseline RT threshold.

**Figure 5:**
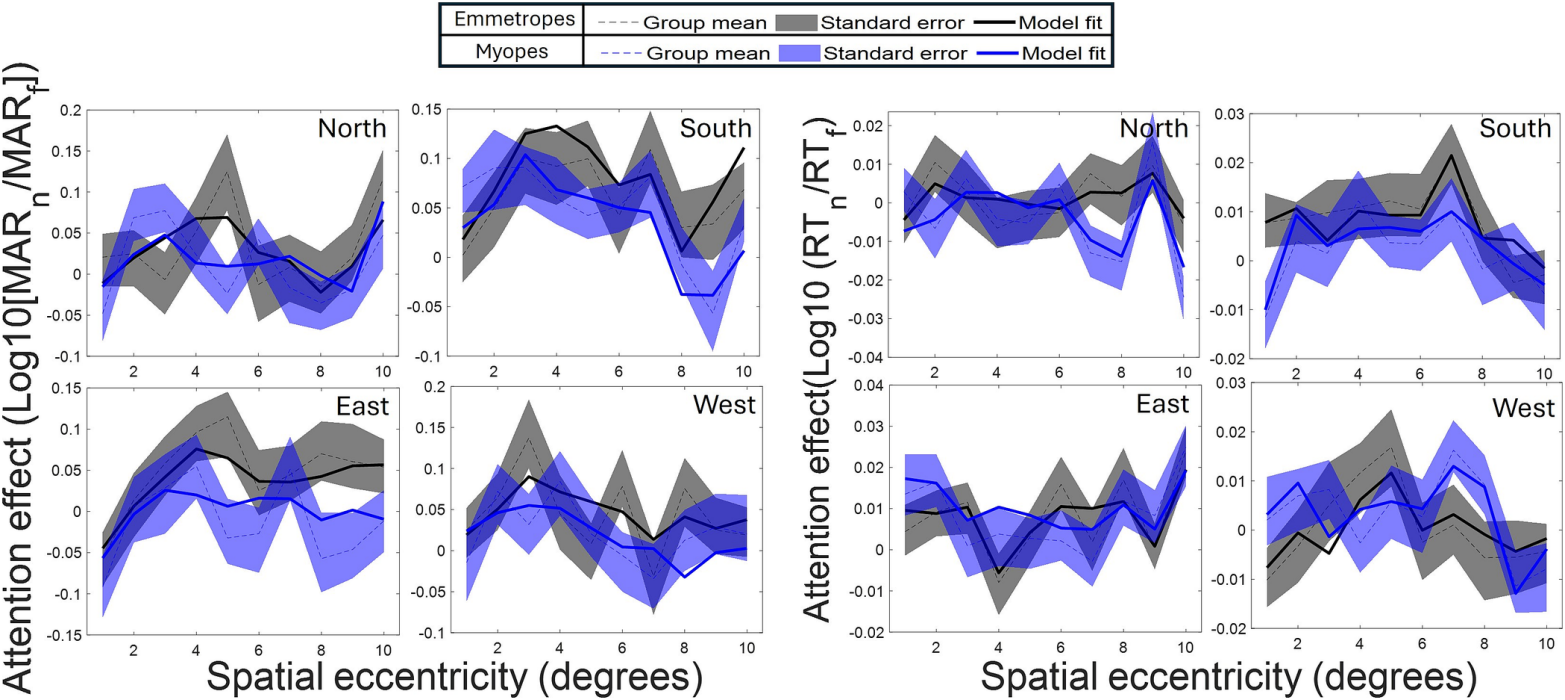
Adjusted model fits. Acuity: Comparison of group-averaged attention effects (dotted line) with model-predicted trajectories (Model 1, full line) across all polar coordinates and target orientations. RT: Same analysis for reaction time. *Grey/purple lines*: Emmetropes/myopes. *Dashed area*: ± SE.

The reduced model confirmed several observations of attention modulation of acuity, including the presence of a curved attentional window (linear eccentricity terms: *β* = 0.005102, SE = 0.002004, *p* < 0.011; quadratic eccentricity terms: *β* = −0.003559, SE = 0.000827, *p* < 0.001; cubic eccentricity terms: *β* = −0.002132, SE = 0.000922, *p* < 0.05) with a peak around 4 degrees from the fovea (see Figures 2, 4); the dependence of attention gain on the polar coordinate with a stronger attention gain in the South position compared to the other polar coordinates (East quadrant: *β* = 0.009520, SE = 0.003028, *p* = 0.002; West quadrant: *β* = 0.005474, SE = 0.003030, *p* < 0.1, North quadrant: *β* = −0.005978, SE = 0.003132, *p* < 0.1); the deficit of attention modulation in myopes with reduced attention-related modulations in RT along the horizontal meridian (Near-emmetropes, East quadrant: *β* = −0.008764, SE = 0.004174, *p* < 0.05; West quadrant: *β* = −0.011743, SE = 0.004174, *p* = 0.005; North quadrant: *β* = −0.000353, SE = 0.004317, *p* > 0.1). In contrast, there was no manifest alteration of the profile of RT modulations by refractive status, rather there was a 5-fold decrease in the gain magnitude of attention modulations for RT compared to acuity, highlighting the reduced influence of the spatial attentional manipulation on RT in the study. This reduced influence is likely due to the stringent temporal constraints imposed on processing time to participant (<800 ms), which may have forced a narrowing of attention around the onset of the target under neutral as well as focused conditions. A close fit of the average attention enhancement with the data was observed (see Figure 5), indicating that the reduced model can explain attention enhancement and its variation across polar coordinates and refractive status.

## 5 Discussion

This study provides the first detailed map and model of attention distribution in myopes and emmetropes (Figure 4), revealing a subtle pattern of attention-related modulations across the visual field, with significant refractive-related attentional differences at the centre of vision. Reduced attentional modulation was observed in myopes for both acuity and RTs compared to emmetropes, revealing a limit of attention focusing in myopes compared to emmetropes. The most striking finding is a restructuration of the enhancement of visual field acuity in myopes, validating the long-held assumption of a spatial narrowing of attention efficiency (Turatto et al., 1999; Mascetti et al., 2001; McKone et al., 2008; Kerber et al., 2016).

With respect to acuity resolution, the primary outcome of this study, both myopes and emmetropes displayed spatial attention-related modulations in acuity that varied in a cubic manner across the visual field. This modulation begins with an increase in attention in the foveal regions, followed by a decline in the parafovea, and concludes with a second enhancement in the perifovea (Figures 2, 4). This profile is reminiscent of previous spatial mapping of spatiotemporal attention, showing multiple, localized attentional enhancement in the fovea (around 0°), parafovea (around 2–3°), and perifovea (around 8–10°) of similar attentional magnitude (Koenig-Robert and Van-Rullen, 2011). It also closely aligned with the proposed “Mexican hat” distribution of attention (Müller et al., 2005; Hopf et al., 2006; Downing, 1988), assuming a progressive decline of attention with distance followed by inhibition.

The study also brings new insights into previous accounts of attentional windows (Yeshurun, 2019) by showing the significant dependence of attention enhancement on meridional position and refractive status, two potential sources of variation in spatial mapping. With respect to meridional dependence, the findings revealed greater attention modulation at the inferior retina, as compared to other meridians, in agreement with previous attention studies (Intriligator and Cavanagh, 2001; Fuller et al., 2008), demonstrating the presence of asymmetry in attention enhancement. Furthermore, the results revealed that attentional enhancements were lower and increasingly reduced with retinal eccentricity in myopes when compared with emmetropes. Based on our model (Figure 4), this linear reduction causes (i) narrowing of the first, central attentional locus in myopes and (ii) inhibitory surrounds in the profile of attention modulation of acuity in myopes.

This alteration in the profile of the spatial attentional window supports the hypothesis formulated in previous research that myopes may deploy their attention over a narrower region of space (Turatto et al., 1999; Mascetti et al., 2001; McKone et al., 2008; Kerber et al., 2016). It is expected that such alterations could significantly recompose the perception of space (Suzuki and Cavanagh, 1997) [and maybe of time (Tse et al., 2004; Poggel et al., 2006)]. A narrowing of attention could be beneficial to enhance blurry visual inputs during uncorrected vision, and may thus reflect a progressive adjustment of neural processing efficiency to the emergence of refractive errors under episodes of uncorrected myopia, favoring central regions of processing over peripheral ones. This would provide a flexible mechanism to cope with episodic, yet regular demands including near reading, without affecting neural responses to unconstrained (far) visual tasks, in contrast to more automatic, invariant adjustments like adaptation. This implies that individual attentional windows can be calibrated according to the level of refractive errors. The weak correlation observed at the attention peak of 5° suggests other factors, such as the onset timing of myopia and the duration of exposure to uncorrected blur, influence the degree of attentional alteration. Although whether the narrowing of attentional enhancement reflects a reduction in attention level/extent remains to be determined, consistent predominance of attention modulations (both acuity and RT) across spatial eccentricities in emmetropes, as compared to myopes, suggests a general deficit in attention in myopes. Further work is necessary to determine whether attentional narrowing reflects a redistribution of attention allocation across the visual field (e.g., superior foveal processing) or a general attentional deficit mediated by receptive field constraints.

Alternatively, it is also clearly plausible that attention deficits precede the onset of myopia. In this context, an effective, rapid *attentional* deblurring could neutralize the need for myopic ocular compensatory responses, addressing the question of whether participants developing myopia may suffer from anomalous attentional compensation to blur. A potential cause of the alteration of attention in myopes could be chronic stress, which appears to impair attention control (Liu et al., 2020), and is a factor recently proposed to be implicated in the epidemic of myopia development (Katz and Berlin, 2014; Lin et al., 2024). As a matter of fact, various studies showed that induced stress (e.g., when individuals struggle to meet task demands) can cause attention narrowing (Staal, 2004). Given that the attention networks recruit several cortical areas proximal to brain regions involved in the control of ocular function, changes in attention could act on changes in ocular (accommodative or pupillary) responses, and more downstream, retinal signaling pathways via feedback neuronal connections. Thus, a recent study (Su et al., 2024) suggested that manipulation of attention by visual crowding may influence short-term eye changes linked to myopia. Furthermore, recent neuroimaging studies showed evidence of important differences in the neural networks in high myopia (Ji et al., 2022; Cheng et al., 2020; Zhang et al., 2020; Zhai et al., 2016; Huang et al., 2016), with changes in the activity of the default mode network (increased functional connectivity (FC) in the posterior cingulate gyrus/precuneus and decreased FC in the left medial prefrontal cortex) (Zhang et al., 2020), dorsal attention network (increased FC in the left inferior parietal gyrus of DAN (Ji et al., 2022), higher amplitude of low-frequency fluctuation in the left inferior parietal lobule (Huang et al., 2016)), executive control network (increased FC of left middle frontal gyrus, left inferior parietal gyrus, and left medial superior frontal gyrus (Liang et al., 2025)), bilateral midcingulate cortex (Cheng et al., 2020; Huang et al., 2016) in high myopes as well as between neural networks [e.g., connection between ventral attention and frontoparietal control networks (Zhai et al., 2016)]. These might point to a complex remodeling of neural processes in progressing myopes, of which the origin and development require attention to clarify the dynamic link between myopia, attentional and cognitive functioning. Thus, stimulation targeting neural loci involved in the focus of attention might provide an interesting method for myopia management. Current methods for mapping attention distribution profiles are time-consuming, limiting research investigation. Therefore, a simplified version of the test needs to be developed for extensive clinical investigations.

## 6 Limitations and considerations

This study has limitations. First, refractive errors could not be fully compensated due to the ongoing challenges in reducing the eye’s peripheral aberrations (Marcos et al., 2022) outside the isoplanatic patch of the eye. While the impact of peripheral refraction on attentional focus is currently unknown, previous studies indicate that differences in relative peripheral refraction between emmetropes and low to moderate myopes slowly increase with eccentricity (Shen et al., 2018), averaging only about 1 diopter at 10° eccentricity. Therefore, this level of variation is likely too subtle to account for the differences in attentional profiles between myopes and emmetropes, particularly given the decline in visual sensitivity to refractive errors with increasing eccentricity (Anderson, 1996). Second, the influence of the time course of the deployment of endogenous attention was not manipulated in the study design. Nevertheless, it has been shown that the time component may only influence the magnitude of attention rather than its spatial profile (Koenig-Robert and Van-Rullen, 2011). Given the observed refractive-related attentional differences, it would be also interesting to examine whether different participants exhibit distinct temporal attentional dynamics, in relation to the size of their attentional focus, over both short-term and longer time scales (For instance, it is possible that deficits of the magnocellular pathway in myopes (Kuo, 2009) could curb the temporal attentional deployment of attention in myopes in a spatially selective manner.) Third, the spatial test was restricted to the central vision regions and cardinal orientation, ignoring a possible dependence of the profile of attention to the level of uncertainty set by the visual field boundaries of the stimulation. Fourth, the attention distribution map assumed a fixed attention focus, rather than variable focus, raising the question of the extent to which participants are capable to precisely spatially allocating their endogenous attention. For instance, a variable focus could cause shift in the center of focus of attention with participant strategies that would cause a reduction of the peak of attention and enlarged width. Fifth, the study assumed a remodeling of endogenous attention with mild to regular myopia, but changes in the automaticity of attention remain to be explored. Indeed, it is important to note that participants with hyperopia, high myopia, and astigmatism were not included, limiting the generalizability of the findings to these refractive error types and severities. Besides, this study employed a relatively small sample size, which can restrict the detection of subtle effect, and thus a larger scale studies will be needed in the future to clarify how different refractive patterns and environmental exposures influence the development of attention.

In addition to the limitation described above, there are methodological differences with previous mapping of attention that are worthy of attention. First, our measure of attention effect was primarily acuity resolution for a suprathreshold stimulus, whereas, in other mapping of spatial attention, contrast sensitivity and RTs were used as a measure of attention. RT measures may be subject to the influence of motor planning, whilst contrast sensitivity measurement needs to account for dependence on the spatio-temporal frequency content of the stimuli. Although acuity is a valuable measure to assess the benefit of attention, the dependence on the neural pathway (e.g., parvocellular versus magnocellular) of attention efficiency could limit an assessment of attention level per se with refractive errors across the visual field. Secondly, the study design used manipulation of spatial uncertainty, rather than cue-based attention, avoiding the pop-up interference of the cue. Third, endogenous attention was sustained to the tested spatial location throughout the block of trials, allowing attention to be fully deployed during the trials, as compared with an interleaved design with alternating attention conditions. Fourth, the study distinguished two additional sources of attentional differences, i.e., refractive errors and angular position, allowing increased accuracy of mapping of spatial attention-related modulation. The presence of the radial grid acted as a placeholder, which allowed participants to anchor their attention along the horizontal and vertical orientations. Fifth, attention was tested monocularly (using the dominant eye) compared with binocular viewing employed in other studies, possibly permitting preferential activation of the attentional hemispheric systems in the contralateral hemisphere (Roth et al., 2002).

## 7 Conclusion

This study modeled for the first time the spatial profile of spatial attention-related modulations in myopes compared to emmetropes, highlighting significant differences in the structure of attention between near emmetropia and mild to regular myopia and providing strong support for a potential narrowing of attention to the foveal regions in myopes. These results suggest a potential remodeling of attention with the development of mild to regular myopia, which warrants further investigation, and may open the path for new myopia treatment methods via the brain.

## Data Availability

The raw data supporting the conclusions of this article will be made available by the authors, without undue reservation.

## Supplementary material

Supplementary table 1 Fixed effects of the full model 1 of attention-related modulations in acuity.

Supplementary table 2 Full model of attention-related modulations in RT.
