# Supplementary material for: Eye-brain connection: an altered profile of spatial attention in myopia
Source: Front Neurosci. 2025 May 23;19:1593463. doi: 10.3389/fnins.2025.1593463 (PMC12141335; doi:10.3389/fnins.2025.1593463)
Supplement: Supplementary table 1 — Fixed effects of the full model 1 of attention-related modulations in acuity. [file Table_1.docx]

| **Source** | **Numerator df** | **Denominator df** | **F** | **P-value** |
| --- | --- | --- | --- | --- |
| Intercept | 1 | 33.437 | 85.632 | 0.000 |
| Baseline neutral acuity | 1 | 1436.763 | 552.187 | 0.000 |
| Polar coordinate | 3 | 358.470 | 8.176 | 0.000 |
| Orient | 1 | 185.494 | 0.384 | 0.536 |
| Refractive status | 1 | 20.647 | 1.393 | 0.251 |
| Eccentricity_sq | 1 | 413.477 | 13.935 | 0.000 |
| Polar coordinate * Orient | 3 | 416.978 | 0.605 | 0.612 |
| Polar coordinate * Refractive status | 3 | 353.028 | 0.597 | 0.617 |
| Polar coordinate * Eccentricity_sq | 3 | 861.229 | 0.423 | 0.737 |
| Orient * Refractive status | 1 | 185.399 | 0.001 | 0.976 |
| Orient * Eccentricity_sq | 1 | 508.630 | 0.340 | 0.560 |
| Refractive status * Eccentricity_sq | 1 | 413.484 | 0.067 | 0.796 |
| Polar coordinate * Orient * Refractive status | 3 | 416.526 | 0.886 | 0.448 |
| Polar coordinate * Orient * Eccentricity_sq | 3 | 951.442 | 0.849 | 0.467 |
| Polar coordinate * Refractive status * Eccentricity_sq | 3 | 860.796 | 1.308 | 0.270 |
| Orient * Refractive status * Eccentricity_sq | 1 | 509.700 | 0.217 | 0.642 |
| Polar coordinate * Orient * Refractive status * Eccentricity_sq | 3 | 951.908 | 1.993 | 0.113 |
| Eccentricity | 1 | 414.517 | 149.561 | 0.000 |
| Polar coordinate * Eccentricity | 3 | 772.485 | 0.966 | 0.408 |
| Orient * Eccentricity | 1 | 449.893 | 4.646 | 0.032 |
| Refractive status * Eccentricity | 1 | 363.978 | 5.512 | 0.019 |
| Polar coordinate * Orient * Eccentricity | 3 | 859.467 | 0.590 | 0.621 |
| Polar coordinate * Refractive status * Eccentricity | 3 | 772.167 | 0.250 | 0.862 |
| Orient * Refractive status * Eccentricity | 1 | 447.714 | 0.163 | 0.686 |
| Polar coordinate * Orient * Refractive status * Eccentricity | 3 | 859.463 | 0.539 | 0.656 |
| Eccentricity_cub | 1 | 869.656 | 15.436 | 0.000 |
| Polar coordinate * Eccentricity_cub | 3 | 1329.167 | 1.397 | 0.242 |
| Orient * Eccentricity_cub | 1 | 988.717 | 5.562 | 0.019 |
| Refractive status * Eccentricity_cub | 1 | 868.582 | 1.322 | 0.251 |
| Polar coordinate * Orient * Eccentricity_cub | 3 | 1379.127 | 0.258 | 0.856 |
| Polar coordinate * Refractive status * Eccentricity_cub | 3 | 1329.738 | 0.059 | 0.981 |
| Orient * Refractive status * Eccentricity_cub | 1 | 987.514 | 0.301 | 0.583 |
| Polar coordinate * Orient * Refractive status * Eccentricity_cub | 3 | 1379.482 | 0.712 | 0.545 |

Table 1. Fixed effects of the full model of attention-related modulations in acuity.
