# Supplementary material for: Eye-brain connection: an altered profile of spatial attention in myopia
Source: Front Neurosci. 2025 May 23;19:1593463. doi: 10.3389/fnins.2025.1593463 (PMC12141335; doi:10.3389/fnins.2025.1593463)
Supplement: Supplementary table 2 — Full model of attention-related modulations in RT. [file Table_2.docx]

| **Source** | **Numerator df** | **Denominator df** | **F** | **P-value** |
| --- | --- | --- | --- | --- |
| Intercept | 1 | 164.119 | 933.437 | 0.000 |
| Baseline neutral RT | 1 | 1455 | 1409.737 | 0.000 |
| Polar coordinate | 3 | 490.084 | 4.626 | 0.003 |
| Orient | 1 | 246.016 | 0.551 | 0.459 |
| Refractive status | 1 | 18.944 | 0.606 | 0.446 |
| Eccentricity_sq | 1 | 490.304 | 18.697 | 0.000 |
| Polar coordinate * Orient | 3 | 611.133 | 0.905 | 0.438 |
| Polar coordinate * Refractive status | 3 | 488.383 | 1.778 | 0.150 |
| Polar coordinate * Eccentricity_sq | 3 | 1059.813 | 0.844 | 0.470 |
| Orient * Refractive status | 1 | 245.385 | 0.019 | 0.891 |
| Orient * Eccentricity_sq | 1 | 647.938 | 0.008 | 0.928 |
| Refractive status * Eccentricity_sq | 1 | 487.595 | 0.912 | 0.340 |
| Polar coordinate * Orient * Refractive status | 3 | 611.413 | 0.249 | 0.862 |
| Polar coordinate * Orient * Eccentricity_sq | 3 | 1174.033 | 0.839 | 0.473 |
| Polar coordinate * Refractive status * Eccentricity_sq | 3 | 1055.551 | 0.103 | 0.958 |
| Orient * Refractive status * Eccentricity_sq | 1 | 647.720 | 0.001 | 0.971 |
| Polar coordinate * Orient * Refractive status * Eccentricity_sq | 3 | 1173.242 | 0.274 | 0.844 |
| Eccentricity | 1 | 430.504 | 3.058 | 0.081 |
| Polar coordinate * Eccentricity | 3 | 973.506 | 1.310 | 0.270 |
| Orient * Eccentricity | 1 | 575.070 | 1.829 | 0.177 |
| Refractive status * Eccentricity | 1 | 429.483 | 2.843 | 0.092 |
| Polar coordinate * Orient * Eccentricity | 3 | 1096.256 | 0.511 | 0.675 |
| Polar coordinate * Refractive status * Eccentricity | 3 | 974.276 | 0.094 | 0.963 |
| Orient * Refractive status * Eccentricity | 1 | 575.490 | 0.433 | 0.511 |
| Polar coordinate * Orient * Refractive status * Eccentricity | 3 | 1095.804 | 3.270 | 0.021 |
| Eccentricity_cub | 1 | 969.404 | 5.836 | 0.016 |
| Polar coordinate * Eccentricity_cub | 3 | 1417.987 | 2.000 | 0.112 |
| Orient * Eccentricity_cub | 1 | 1127.116 | 7.477 | 0.006 |
| Refractive status * Eccentricity_cub | 1 | 969.313 | 3.265 | 0.071 |
| Polar coordinate * Orient * Eccentricity_cub | 3 | 1455 | 0.588 | 0.623 |
| Polar coordinate * Refractive status * Eccentricity_cub | 3 | 1418.730 | 0.163 | 0.922 |
| Orient * Refractive status * Eccentricity_cub | 1 | 1128.994 | 0.956 | 0.328 |
| Polar coordinate * Orient * Refractive status * Eccentricity_cub | 3 | 1455 | 3.860 | 0.009 |

Table 2. Full model of attention-related modulations in RT
